# A systemic review and meta-analysis on the efficacy and safety of ferumoxytol for anemia in chronic kidney disease patients

**DOI:** 10.1080/0886022X.2021.2021237

**Published:** 2022-02-14

**Authors:** Qianwei Zuo, Taizhong Wang, Lirong Zhu, Xiao Li, Qi Luo

**Affiliations:** a Department of Nephrology, People’s Hospital of Qijiang Distract, Chongqing, P.R. China; b Department of Nursing, People’s Hospital of Qijiang Distract, Chongqing, P.R. China; c Department of Hematology, People’s Hospital of Qijiang Distract, Chongqing, P.R. China

**Keywords:** Chronic kidney disease, hemodialysis, hemoglobin, ferritin, adverse event

## Abstract

**Purpose:**

To evaluate the efficacy of ferumoxytol, relative to conventional iron supplement formulations, on hemoglobin levels, ferritin level, and adverse event incidence in chronic kidney disease patients.

**Methods:**

We performed a systematic search of six academic databases (EMBASE, CENTRAL, Scopus, PubMed, Web of sciences, and MEDLINE), adhering to PRISMA guidelines. We performed a meta-analysis on relevant studies to evaluate the overall influence of ferumoxytol, relative to conventional iron supplement formulations, on hemoglobin levels, ferritin level, and treatment related treatment emergent adverse events (TEAEs) incidence in chronic kidney disease patients.

**Results:**

Seven eligible studies were identified from a total of 1397 studies. These studies contained data on 3315 participants with chronic kidney disease (mean age: 59.2 ± 4.6 years). A meta-analysis revealed that ferumoxytol administration had positive effects on hemoglobin levels (Hedge’s g statistic: 0.51) and ferritin level (0.88), transferrin saturation (0.39). Besides, we also report reduced incidence of treatment related TEAEs (−0.24) for patients consuming ferumoxytol as compared conventional iron supplement formulations.

**Conclusions:**

This meta-analysis provides preliminary evidence that ferumoxytol use exerts beneficial effects on the overall hematological outcomes in patients with chronic kidney disease. This study also reports improved treatment related safety profile for ferumoxytol when compared with conventional iron formulations. The findings from this study can have direct implications in forming best practice guidelines for managing anemia in patients with chronic kidney disease.

## Introduction

Chronic kidney disease is one of the most common renal disorders worldwide [1]. According to the National Kidney Foundation, it is characterized as a pathological condition where glomerular filtration rate drops below ≤ 60 mL/min/1.73 m^2^ [2]. Epidemiological studies have widely reported high prevalence rates of around 10–13% for chronic kidney disease amongst the general population [1,3]. Moreover, the World Health Organization notes that almost 1.1 million deaths occur worldwide every year due to chronic kidney disease [4].

Researchers have linked the presence of anemia in chronic kidney disease with worse prognostic outcomes in terms of mortality and long-term quality of life [5,6]. Erythrocytes are produced through erythropoiesis, a process typically reliant on sufficient levels of a glycoprotein-cytokine such as kidney-produced erythropoietin [7]. However, the extensive damage to nephrotic structures caused by chronic kidney disease, especially within the cortex and medulla, hinders erythropoietin manufacturing [8] and subsequent red blood cell production [9]. In addition, elevated hepcidin levels due to nephrotic inflammation can also instigate anemia by disrupting iron transport and metabolism, as well as by inhibiting erythropoiesis stimulators [10,11]. Finally, increased blood loss due to hemodialysis, increased uremic-inhibitor levels, and reduced erythrocyte lifespan have all been identified as additional factors that cause or aggravate anemia [12–14].

Currently, anemia is managed in chronic kidney disease patients using semi-synthetic poly-glucose sorbitol carboxymethyl ether shell-coated super-paramagnetic iron-oxide nanoparticles such as ferumoxytol [15,16]. The carbohydrate shell helps to isolate the super-paramagnetic iron-oxide nanoparticle from blood plasma, which facilitates particle uptake by reticuloendothelial system macrophages [15]. In addition, ferumoxytol has been reported to contain the lowest concentrations of ultra-filtrable free iron and dialyzable free iron [16,17]. Ferumoxytol is also popular due to its ability to maintain concentration levels during hemodialysis [18]. That said, a consensus on the efficacy of ferumoxytol compared to other iron supplement formulations for managing anemia in chronic kidney disease is still lacking.

A few randomized controlled trials have attempted to evaluate the efficacy of ferumoxytol compared to other iron supplements, such as iron sucrose, for managing chronic kidney disease. Some studies have reported that oral [19] and intravenous [20] administration of ferumoxytol resulted in reduced adverse events. However, others have noted the opposite [21]. Naturally, no meta-analysis currently exists seeking to evaluate the overall influence of ferumoxytol on chronic kidney disease compared to other iron supplement formulations.

Thus, we herein perform a meta-analysis on the current literature in order to summarize the available data on ferumoxytol use for chronic kidney disease. Our investigation looks at hemoglobin levels, ferritin level, transferrin saturation, and treatment related Treatment Emergent Adverse Events (TEAEs) incidence in chronic kidney disease patients administered ferumoxytol compared to those in patients taking conventional iron supplement formulations. Our findings will hopefully raise clinical awareness among nephrologists concerning how to manage and treat chronic kidney disease and its associated complications.

## Methods

This literature review and meta-analysis was performed according to PRISMA (Preferred Reporting Items for Systematic Reviews and Meta-Analyses) guidelines [22].

### Data search strategy

Articles published between January 1950 to October 2020 in six academic databases were searched (MEDLINE, CENTRAL, EMBASE, PubMed, Web of sciences, and Scopus). A sample search strategy for the EMBASE database has been provided in Supplementary file. Furthermore, the reference/bibliography sections of included studies were screened manually to identify additional relevant studies. The screening procedure adhered to the following inclusion criteria:

- Studies must have evaluated hemoglobin levels, ferritin levels (i.e., measures the level of protein ferritin that stores iron in the cells), and treatment related TEAEs incidence in chronic kidney disease patients receiving ferumoxytol.
- Studies must involve human subjects.
- Studies must be either randomized controlled trials, quasi-randomized controlled trials, controlled clinical trials, prospective observational trials with control groups, or retrospective trials.
- Studies must have been published in peer-reviewed academic journals or as part of scientific conference proceedings.
- Studies must have been published in the English language.

Database and study screening was performed by two independent reviewers. A third independent reviewer served as arbitrator in case of dispute. During screening, we extracted the following information from included studies: author information, descriptive data, country in which the study was conducted, study type, drug administered, dosage regimen follow-up, hemoglobin levels, ferritin level, and adverse event outcomes. For studies where quantitative data was not publically available, we made multiple attempts to contact the study authors to gain access to the relevant data.

### Quality assessment

We used Cochrane’s risk of bias assessment tool for randomized controlled trials [23] to assess bias risk in each included individual trial. This tool assesses the possibility of bias in the form of inadequate randomization, selective reporting, concealed allocation, blinding of outcomes, and incomplete data. Methodological quality was appraised by two independent reviewers, with, again, a third reviewer serving as arbitrator if necessary.

### Data analysis

Within-group meta-analysis was performed using CMA software (Comprehensive Meta-analysis version 2.0) [24]. The meta-analysis was performed based on a random-effects model [25]. We estimated the weighted effect size (Hedge’s g statistic) and assessed heterogeneity among the studies (*I*^2^ statistic). The thresholds used to interpret heterogeneity were as follows: *I*^2^ between 0% and 25%: negligible heterogeneity; 25–75%: moderate heterogeneity; ≥75%: substantial heterogeneity [26]. We examined data distribution and analyzed hemoglobin, ferritin levels, and adverse event incidence between study groups receiving ferumoxytol and those receiving iron supplement formulations. We reported prevalence rates, 95% confidence intervals, levels of significance, and heterogeneity. We also analyzed publication bias using Duval and Tweedy’s trim and fill procedure [27], a method that evaluates whether the overall effect would shift if apparent bias were to be removed. The significance level for this study was determined to be 5%.

## Results

**Figure 1.**
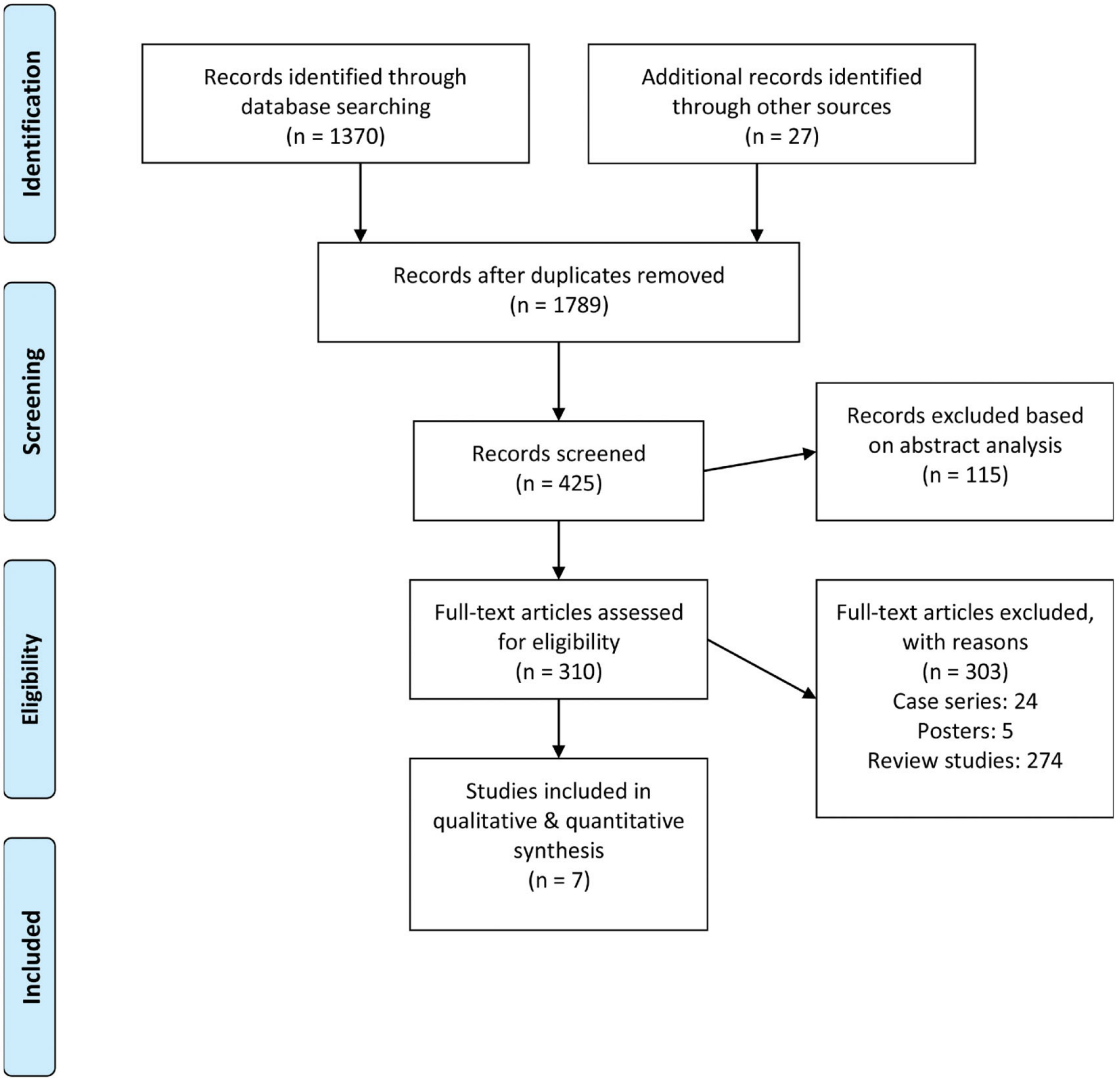
PRISMA flowchart detailing study identification and inclusion.

The systemic literature search performed on six databases identified 1370 candidate studies, while bibliography screening revealed an additional 27 study candidates (Figure 1). After applying inclusion criteria, a total of seven studies remained, all of which were randomized controlled trials [16,19–21,28–30]. The extracted data from these seven studies is found in detail in Table 1.

**Table 1.** Details of the included studies.

Study	Patient status	Type of study	Drugs and strength	Sample descriptive	Age (M ± S.D., years)	Follow- up	Hemoglobin difference (g/dL)	Ferritin (ng/mL)	TSAT (%)	Treatment related treatment emergent adverse events (n)
Macdougall, Strauss et al. (2019)	HD	Randomized controlled trial	Exp: Ferumoxytol IV: 2 × 510 mg Ct: Iron Sucrose IV: 10 × 100mg	Exp: 196 (82 F, 114 M) Ct: 97 (40 F, 57 M)	Exp: 59.3 ± 14.1 Ct: 57.6 ± 13.6	35-days	Exp: 0.5 Ct: 0.4	–	–	Exp: 14 Ct: 4
Strauss, Dahl et al. (2016)	ND	Randomized controlled trial	Exp: Ferumoxytol IV: 2 × 510 mg Ct: IV injection or infusion of iron sucrose 200 mg (10 mL) on Day 1 and on 4 other nonconsecutive days over a 14-day period, for a total cumulative dose of 1 g	Exp: 406 Ct: 199	Exp: 50.1 ± 15.7 Ct: 52.8 ± 16	35-days	Exp: 3.3 ± 1.3 Ct: 2.8 ± 0.98	Exp: 98.3 ± 118.9 Ct: 96.4 ± 185.2	Exp: 22.9 ± 21.7 Ct: 19.1 ± 14.7	Exp: 206 Ct: 141
	HD		Ct: slow IV injection or IV drip infusion of 100 mg of iron sucrose on day 1 and at the following 9 consecutive hemodialysis sessions for a total cumulative dose of 1g	Exp: 80 Ct: 82		35-days	–	Exp: 788.4 ± 427.2 Ct: 618.9 ± 355.4	Exp: 28.7 ± 8.8 Ct: 27.9 ± 15.8	
Macdougall, Strauss et al. (2014)	ND	Randomized controlled trial	Exp: Ferumoxytol IV: 2 × 510 mg Ct: Iron Sucrose IV: 10 × 100 mg	Exp: 80 (41 F, 39 M) Ct: 82 (39 F, 43 M)	Exp: 62 ± 15 Ct: 63 ± 15	21-days	Exp: 0.8 ± 0.1 Ct: 0.7 ± 0.1	Exp: 631 Ct: 456	Exp: 27.5 ± 8 Ct: 25.5 ± 12.7	Exp: 86 Ct: 161
Lu, Cohen et al. (2010)	ND	Randomized controlled trial	Exp: Ferumoxytol IV: 2 × 510 mg Ct: Oral iron 200 mg/day	Exp: 228 (−) Ct: 76 (−)	–	35-days	Exp: 0.82 ± 1.24 Ct: 0.16 ± 1.02			Exp: 9 Ct: 25
				Exp: 226 (−) Ct: 77 (−)			Exp: 1.22 ± 1.25 Ct: 0.52 ± 0.98			
	HD			Exp: 114 (−) Ct: 116 (−)			Exp: 1.02 ± 1.13 Ct: 0.46 ± 1.06			
Provenzano, Schiller et al. (2009)	HD	Randomized controlled trial	Exp: Ferumoxytol IV: 2 × 510 mg Ct: Oral iron 200 mg/day	Exp: 114 (57 F, 57 M) Ct: 116 (43 F, 73 M)	Exp: 59.5 ± 14.3 Ct: 60.8 ± 13	21-days	–	Exp: 356.6 ± 247.1 Ct: −37.5 ± 106.9	Exp: 6.2 ± 12.1 Ct: 1.3 ± 8.8	Exp: 54 Ct: 64
Singh, Patel et al. (2008)	ND	Randomized controlled trial	Exp: Ferumoxytol IV: 2 × 510 mg Ct: Placebo	Exp: 362 (195 F, 167 M) Ct: 360 (195F, 165M)	Exp: 64.2 ± 14.5 Ct: 63 ± 15.1	7-days	–	–		Exp: 53 Ct: 42
Spinowitz, Kausz et al. (2008)	ND	Randomized controlled trial	Exp: Ferumoxytol IV: 2 × 510 mg Ct: Oral iron 200 mg/day	Exp: 228 (134 F, 94 M) Ct: 76 (52 F, 24 M)	Exp: 65.1 ± 14.3 Ct: 63.7 ± 11.1	35-days	Exp: 0.82 ± 1.24 Ct: 0.16 ± 1.02	Exp: 381.7 ± 278.6 Ct: 6.9 ± 60.1	Exp: 9.8 ± 9.2 Ct: 1.3 ± 6.4	–

M: Mean; S.D: Standard deviation; IV: Intravenous; Exp: Experimental group; Ct: Control group; ND: Non-dialysis; HD: Hemodialysis; TSAT: Transferrin saturation.

### Participant information

The seven studies contained data from a total of 3315 (878 female, 833 male, 1323 not available) patients with chronic kidney disease. Two studies did not provide patient gender information [19,30] A total of 2034 (509 female, 471 male, 886 not available) patients received ferumoxytol, 1281 (369 female, 362 male, 437 not available) received iron supplements or placebo.

Average participant age was 59.2 ± 4.6 years. The average age of ferumoxytol-receiving patients was 59 ± 5.3 years, while the comparator group average age was 59.4 ± 4.3 years.

### Randomized controlled trial quality assessment

**Figure 2.**
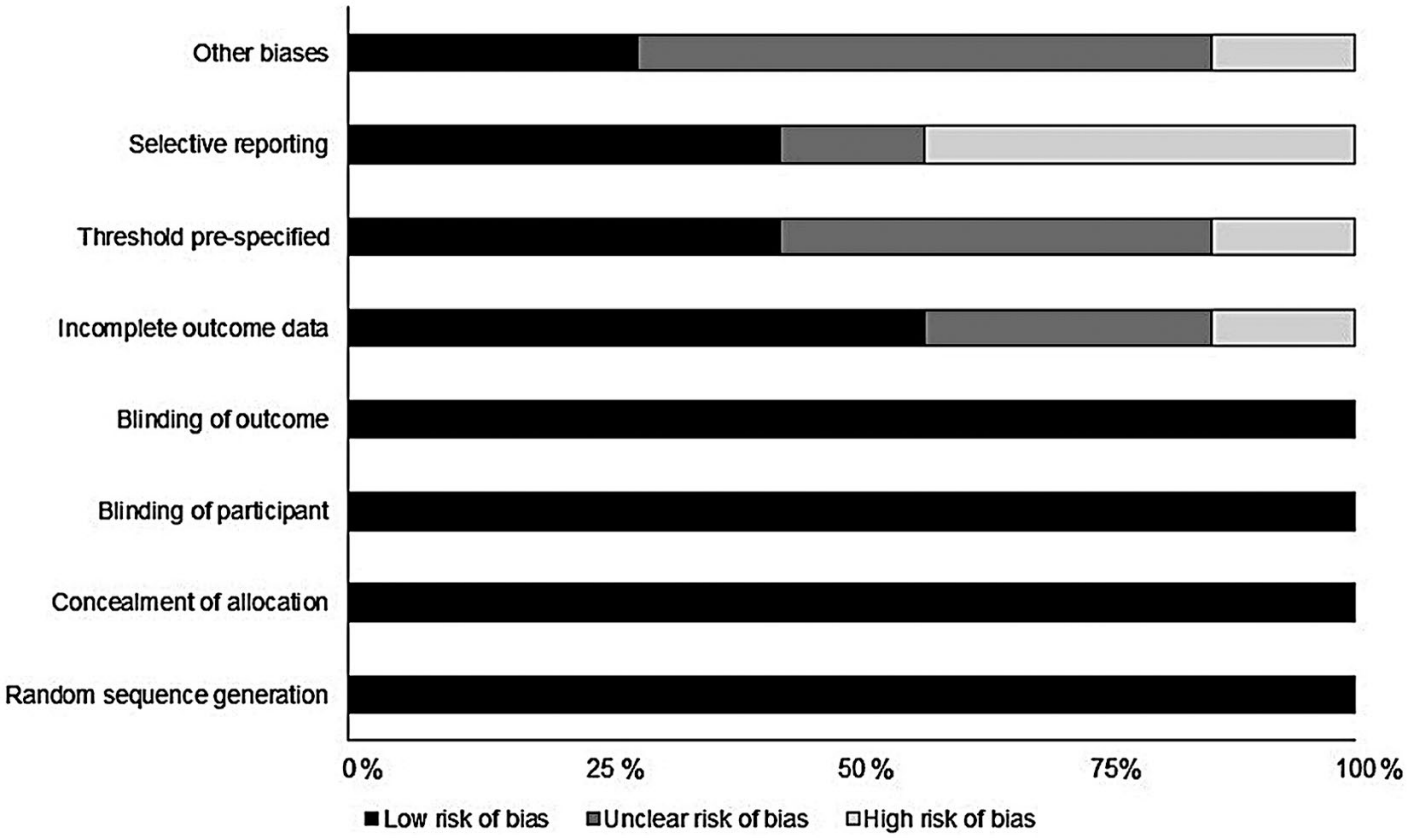
Bias risk for included trials based on the Cochrane risk of bias assessment.

The methodologies of the included randomized controlled trials were assessed for bias risk (Table 2, Figure 2). Overall risk was found to be low, with selective reporting most prominent.

**Table 2.** Bias risk within included studies according to the Cochrane risk of bias tool for randomized controlled trials.

	Random sequence generation	Concealment of allocation	Blinding of participant	Blinding of outcome	Incomplete outcome data	Threshold pre-specified	Selective reporting	Other biases
Macdougall et al. (2019)	+	+	+	+	+	−	−	?
Strauss et al. (2016)	+	+	+	+	?	?	?	−
Macdougall et al. (2014)	+	+	+	+	+	+	+	+
Lu et al. (2010)	+	+	+	+	−	?	−	?
Provenzano et al. (2009)	+	+	+	+	+	+	+	+
Singh et al. (2008)	+	+	+	+	+	+	+	?
Spinowitz, Kausz et al. (2008)	+	+	+	+	−	?	−	?

### Publication bias

**Figure 3.**
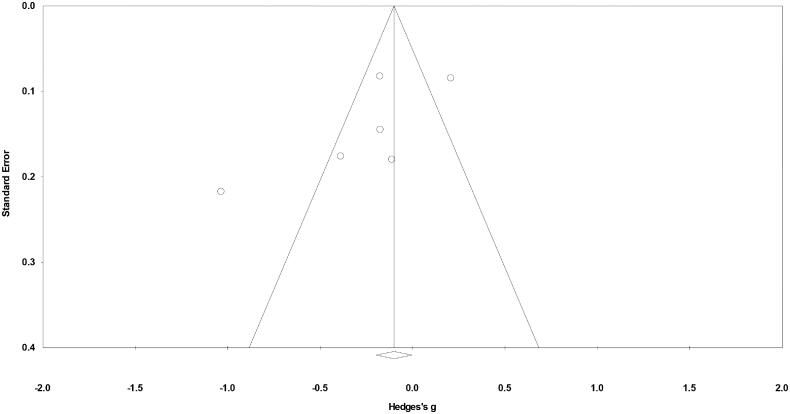
Publication bias determined using the Duval & Tweedy’s trim and fill method.

Duval and Tweedy’s trim and fill method was used to identify missing studies on either side of a funnel plot according to a random effects model. No study was found to be missing on either side of the mean effect. The overall random effects model determined the point estimates and the 95% confidence intervals for the included studies to be −0.24 (−0.52 to 0.03). Application of the trim and fill method did not alter these values (Figure 3).

## Meta-analysis report

### Hemoglobin level

**Figure 4.**
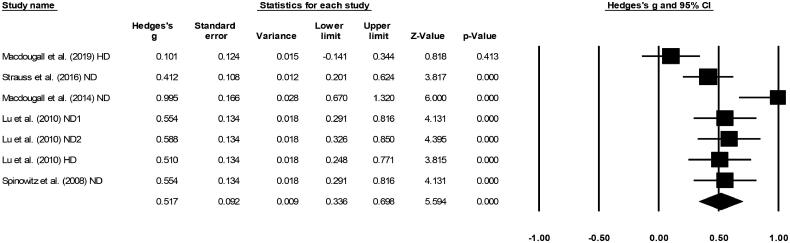
Forest plot for studies evaluating hemoglobin levels in chronic kidney disease patients receiving either ferumoxytol or iron supplements. The overall effect size is presented as black boxes while 95% confidence intervals are presented as whiskers. A negative effect size represents higher hemoglobin levels for patients administered iron supplements, while a positive effect size represents higher hemoglobin levels for patients administered ferumoxytol. (ND: Non-dialysis; HD: Hemodialysis).

Five included studies had reported mean differences in hemoglobin levels [19,20,28–30] A medium positive effect (Hedge’s g statistic: 0.51, Standard error: 0.09, Variance: 0.009, Figure 4) for ferumoxytol was observed (95% C.I: 0.33–0.69, *Z*-value: 5.59, *p* < 0.01), with negligible heterogeneity (*I*^2^: 9.5%).

### Ferritin level

**Figure 5.**
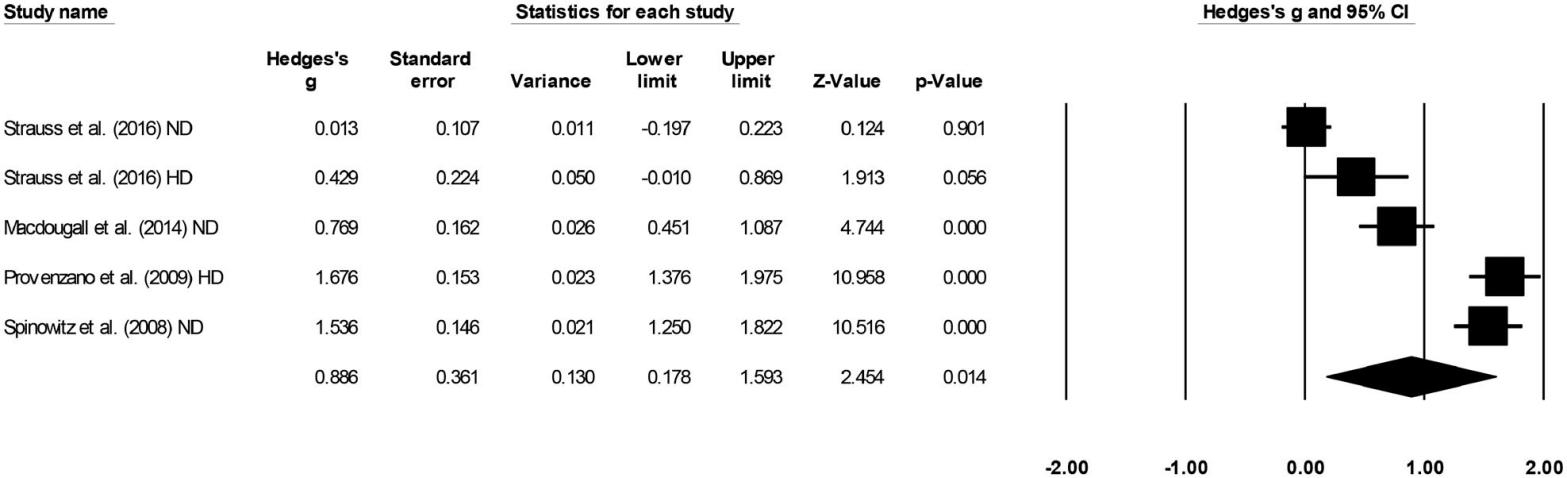
Forest plot for studies evaluating ferritin levels in chronic kidney disease patients receiving either ferumoxytol or iron supplements. The overall effect size is presented as black boxes while 95% confidence intervals are presented as whiskers. A negative effect size represents higher ferritin levels for patients administered iron supplements, while a positive effect size represents higher ferritin levels for patients administered ferumoxytol. (ND: Non-dialysis; HD: Hemodialysis).

Four included studies evaluated ferritin levels [16,28–30]. A large positive effect (Hedge’s g statistic: 0.88, Standard error: 0.36, Variance: 0.13, Figure 5) for ferumoxytol was observed (95% C.I: 0.17–1.59, *Z*-value: 2.4, *p* = 0.01), with no heterogeneity (*I*^2^: 0%).

### Transferrin saturation

**Figure 6.**
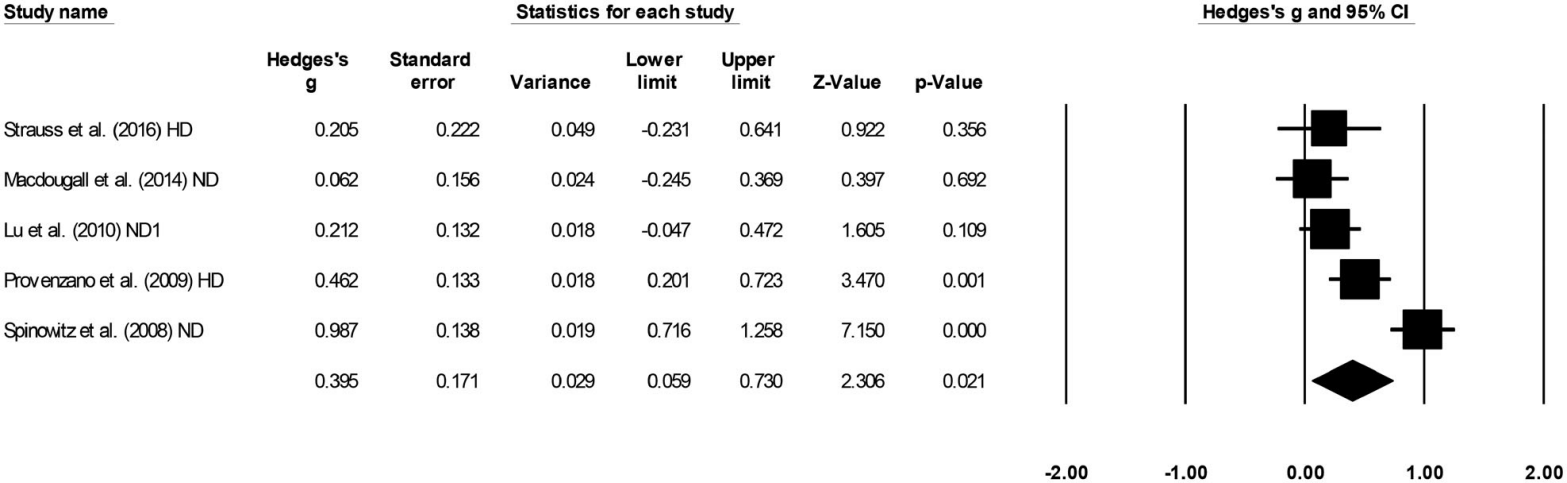
Forest plot for studies evaluating transferrin saturation in chronic kidney disease patients receiving either ferumoxytol or iron supplements. The overall effect size is presented as black boxes while 95% confidence intervals are presented as whiskers. A negative effect size represents higher transferrin saturation for patients administered iron supplements, while a positive effect size represents higher transferrin saturation for patients administered ferumoxytol. (ND: Non-dialysis; HD: Hemodialysis).

Five included studies evaluated transferrin saturation [16,19,28–30]. A medium positive effect (Hedge’s g statistic: 0.39, Standard error: 0.17, Variance: 0.02, Figure 6) for ferumoxytol was observed (95% C.I: 0.05–0.73, *Z*-value: 2.3, *p* = 0.02), with no heterogeneity (*I*^2^: 0%).

### Treatment related TEAEs

**Figure 7.**
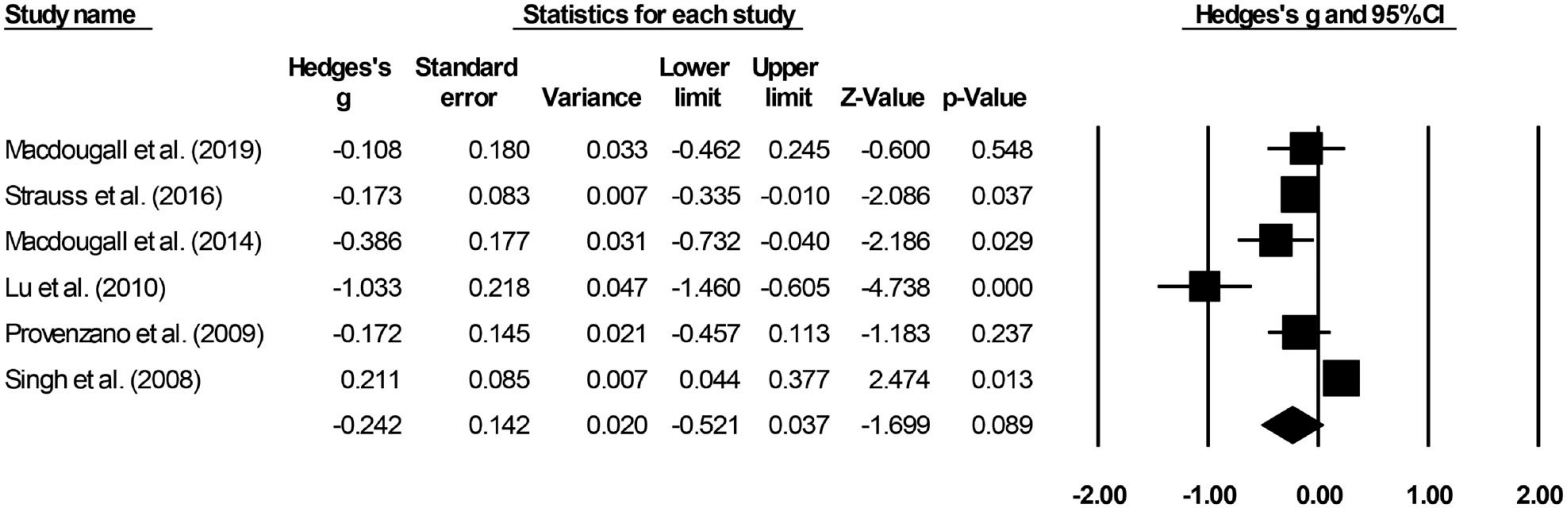
Forest plot for studies evaluating adverse event incidence in chronic kidney disease patients receiving either ferumoxytol or iron supplements. The overall effect size is presented as black boxes while 95% confidence intervals are presented as whiskers. A negative effect size represents more adverse events for patients administered iron supplements, while a positive effect size represents more adverse events for patients administered ferumoxytol.

Six included studies evaluated treatment related TEAEs [19–21,28–30]. A small negative effect (Hedge’s g statistic: −0.24, standard error: 0.14, variance: 0.02 Figure 7) for the iron supplement-receiving patients was observed (95% C.I: −0.52–0.03, *Z*-score: −1.69, *p* = 0.08), with negligible heterogeneity (*I*^2^: 24.1%).

## Discussion

This meta-analysis provides novel evidence regarding the comparative efficacy of ferumoxytol against other conventional iron supplement formulations for managing chronic kidney disease. We observed that ferumoxytol administration was beneficial relative to conventional iron supplement formulations in terms of hemoglobin level management, ferritin level, transferrin saturation and limiting treatment related TEAEs in patients with chronic kidney disease.

Managing chronic kidney disease is highly challenging because of its atypical pathophysiological mechanisms, co-existing morbidities, and manifestation [31,32]. Anemia, one of the most common co-morbidities [33], has been associated with poor prognostic outcome in terms of short- and long-term morbidity and mortality for chronic kidney disease [34–36]. Anemia onset in chronic kidney disease patients is primarily caused by reduced erythropoietin levels and disrupted iron metabolism [14]. Studies have also suggested a direct relationship between anemia severity and chronic kidney disease severity [33]. Ferumoxytol use has intensified in recent decades for managing anemia co-existing with chronic kidney disease for a number of reasons [15,16]. First, ferumoxytol reportedly overcomes reticuloendothelial system blockage caused by chronic kidney disease, thereby ensuring sufficient iron reserve bioavailability for anemia management [37]. Second, the drug possesses a superior pharmaco-kinetic profile, presenting a higher safety profile and permitting daily dosing regimens [15]. Third, ferumoxytol concentrations are maintained even after hemodialysis [18]. Fourth, this compound can enhance hemoglobin levels and ferritin levels relative to conventional iron supplement formulations.

This last point is echoed in our study, where all the included studies reported that ferumoxytol was beneficial relative to conventional iron supplement formulations when it came to mediating hemoglobin levels in chronic kidney disease patients. Provenzano et al. [29], for instance, reported in a phase-III randomized controlled trial that a 510 mg dosage of ferumoxytol increased hemoglobin levels and ferritin levels far more than a 200-mg dosage of oral iron supplementation after 21 and 35 days of administration among 230 chronic kidney disease patients on hemodialysis. The authors also mentioned that ferumoxytol also improved other hematological outcomes such as transferrin saturation, reticulocyte hemoglobin content, and total iron binding capacity. Similarly, Macdougall et al. [28] reported that 1020 mg ferumoxytol in two doses outperformed 1000 mg iron sucrose administered *via* injection/intravenous infusion. Here, ferumoxytol improved hematological outcomes beginning at 14 days until 35 days after administration. In our present meta-analysis, we confirm these findings and report that ferumoxytol administration resulted in a medium positive overall effect on hemoglobin levels (Hedge’s g statistic: 0. 33), and transferrin saturation (Hedge’s g: 0.39) in chronic kidney disease patients. We also reported that ferumoxytol administration resulted in a large positive effect on ferritin level (Hedge’s g: 0.88) in chronic kidney disease patients.

We also attempted to establish whether there were differences in treatment related TEAEs incidence when using ferumoxytol versus other conventional iron supplement formulations. Here, Lu et al., Macdougall et al., Strauss et al., and Provenzano et al. [19,28–30] all reported fewer treatment related TEAEs for ferumoxytol-using patients compared to those given conventional iron supplement formulations. Overall, our meta-analysis reports a decreased treatment related TEAEs effect for ferumoxytol users relative to conventional iron supplement formulation-using chronic kidney disease patients.

This study was hampered by several limitations. First, we were unable to register this meta-analysis in a review repository such as PROSPERO. This may raise some concern regarding the validity of this study [38]. However, we made considerable efforts to register our review with PROSPERO and other such repositories, only to be informed that wait times exceeded 1 year due to the current pandemic crisis. Second, we did not provide a descriptive explanation for the studies study that were excluded. Third, insufficient available data meant that we could not carry out sub-group analyses effectively. Moreover, as we did not observe heterogeneity, we strongly recommend that our results be interpreted with caution and hope that future studies will be able to address these limitations by making more data publicly available.

In conclusion, we herein provide a preliminary summation regarding the comparative efficacy of ferumoxytol versus conventional iron supplement formulations. We note that ferumoxytol elevates hemoglobin levels, transferrin saturation and ferritin levels in patients with chronic kidney disease, compared to conventional iron supplement formulations. Moreover, we report reduced incidence of treatment related TEAEs with ferumoxytol administration.

## Data Availability

The datasets used and/or analyzed during the current study are available from the corresponding author on reasonable request.
